# Loss of daptomycin susceptibility in clinical *Staphylococcus epidermidis* infection coincided with variants in *WalK*

**DOI:** 10.1093/emph/eoaa031

**Published:** 2020-09-07

**Authors:** Nicholas F Brazeau, Kara J Levinson, Asher Schranz, Kara A Moser, Ian Hollis, Prashanth Iyer, Christopher Chien, Amanda Bowen, David van Duin, Anne Lachiewicz, Tessa Andermann, Melissa Jones, Melissa Miller, Jonathan J Juliano, Luther A Bartelt

**Affiliations:** Department of Epidemiology, Gillings School of Global Public Health, University of North Carolina, Chapel Hill, NC 27599, USA; Medical Scientist Training Program, University of North Carolina School of Medicine, Chapel Hill, NC 27599, USA; Department of Pathology and Laboratory Medicine, School of Medicine, University of North Carolina, Chapel Hill, NC 27599, USA; Clinical Microbiology Laboratory, University of North Carolina Health Care, Chapel Hill, NC 27599, USA; Division of Infectious Diseases, Department of Medicine, School of Medicine, University of North Carolina at Chapel Hill, Chapel Hill, NC, 27599, USA; Department of Epidemiology, Gillings School of Global Public Health, University of North Carolina, Chapel Hill, NC 27599, USA; University of North Carolina Health, UNC Eshelman School of Pharmacy, University of North Carolina at Chapel Hill, Chapel Hill, NC, 27599 USA; University of North Carolina Health, UNC Eshelman School of Pharmacy, University of North Carolina at Chapel Hill, Chapel Hill, NC, 27599 USA; Division of Cardiology, University of North Carolina at Chapel Hill, Chapel Hill, NC 27599, USA; Curriculum in Genetics and Molecular Biology, University of North Carolina, Chapel Hill, NC27599 USA; Division of Cardiology, University of North Carolina at Chapel Hill, Chapel Hill, NC 27599, USA; Curriculum in Genetics and Molecular Biology, University of North Carolina, Chapel Hill, NC27599 USA; Division of Infectious Diseases, Department of Medicine, School of Medicine, University of North Carolina at Chapel Hill, Chapel Hill, NC, 27599, USA; Division of Infectious Diseases, Department of Medicine, School of Medicine, University of North Carolina at Chapel Hill, Chapel Hill, NC, 27599, USA; Division of Infectious Diseases, Department of Medicine, School of Medicine, University of North Carolina at Chapel Hill, Chapel Hill, NC, 27599, USA; Clinical Microbiology Laboratory, University of North Carolina Health Care, Chapel Hill, NC 27599, USA; Department of Pathology and Laboratory Medicine, School of Medicine, University of North Carolina, Chapel Hill, NC 27599, USA; Clinical Microbiology Laboratory, University of North Carolina Health Care, Chapel Hill, NC 27599, USA; Department of Epidemiology, Gillings School of Global Public Health, University of North Carolina, Chapel Hill, NC 27599, USA; Division of Infectious Diseases, Department of Medicine, School of Medicine, University of North Carolina at Chapel Hill, Chapel Hill, NC, 27599, USA; Division of Infectious Diseases, Department of Medicine, School of Medicine, University of North Carolina at Chapel Hill, Chapel Hill, NC, 27599, USA

**Keywords:** *Staphylococcus epidermidis*, daptomycin, antibiotic resistance

## Abstract

Daptomycin (DAP) is key in treating multidrug-resistant *Staphylococcus* infections. Diminished susceptibility to DAP is emerging among *Staphylococcus epidermidis* strains although mechanisms for non-susceptibility (NS) remain poorly understood. We report a case of persistent *S. epidermidis* bacteremia in which loss of DAP susceptibility arose during prolonged treatment. Whole genome sequencing identified two mutations, Q371del and P415L, in a single-affected gene, *WalK*, that coincided with the emergence of DAP-NS. Protein modeling of the mutations predicted a disruption of WalK protein configuration. The emergence of mutations in a single-gene during DAP exposure raises concerns in an era of increasingly treatment-resistant infections.

Lay summary: Daptomycin is an important antibiotic for fighting *Staphylococcus* infections. We identified variants in the *WalK* gene that were coincident with resistance in a clinical *Staphylococcus epidermidis* infection. Clinicians, hospital epidemiologists, and microbiology laboratories need to be aware of the potential for the evolution of drug resistance during prolonged daptomycin therapy.

## INTRODUCTION

Coagulase-negative *Staphylococci* (CoNS), including *S. epidermidis*, are capable of biofilm production, and are therefore one of the most common causes of endovascular device-related bloodstream infection (BSI) [1]. *Staphylococcus epidermidis* strains causing infections are increasingly multi-drug resistant (MDR) [2]. Guideline-based management of MDR-*S. epidermidis* endovascular device-related BSI includes parenteral antibiotics (primarily vancomycin) and removal of the device [3]. However, in some settings, such as patients with a left ventricular assist device (LVAD), device removal is often impractical. In these cases—absence of biofilm removal—prolonged courses of antimicrobial therapy may increase the potential for further drug-resistance development.

Daptomycin (DAP), a cyclic lipopeptide, demonstrates concentration-dependent bactericidal activity against *Staphylococcus spp.* [4]. Daptomycin is frequently used as an alternative to vancomycin for the treatment of VAD-related *S. epidermidis* BSI [5–7]. Although more than 98% of CoNS are reported to be susceptible to DAP [8], recent reports raise concern for emerging DAP resistance: (i) there are an increasing number of DAP non-susceptible (DAP-NS) CoNS isolates (minimum inhibitory concentrations [MICs] >2 μg/ml) and (ii) DAP-NS CoNS strains have been recovered from endovascular devices during DAP therapy [9].

Treatment-emergent DAP-NS *S. aureus* has been shown to occur through heterogenous pathways [10]; however, the mechanisms of clinically emergent DAP-NS *S. epidermidis* and other CoNS remain less well defined. One potentially overlapping mechanism across multiple *Staphylococcus spp.* is the environmental sensing apparatus, WalKR (syn: YycG/YycF). *WalK* is a sensor protein kinase and a member of the two-component regulatory system *WalK*/*WalR* (*WalKR*) that regulates genes involved in autolysis, biofilm formation, and cell-wall metabolism/degradation [11]. Increasing sub-inhibitory doses of DAP to a biofilm-producing laboratory *S. epidermidis* strain (ATCCRP62a) resulted in a DAP-NS phenotype via a single-nucleotide mutation in the *WalK* (V500F) [12, 13]. The functional relevance of V500F was demonstrated in an isogenic mutant RP62aWalK^V500F^ with DAP-NS [13]. In a recent report, 6/17 of DAP-NS CoNS were found to have *WalKR* mutations [13]. However, whether *WalKR* mutations are sufficient to result in clinical-emergent DAP-NS CoNS and/or clinical failure has yet to be proven. Furthermore, given the potential for antimicrobial heteroresistance among bacterial sub-populations within a given biofilm, it is unclear to what extent antibiotic-resistant strains emerge through: (i) *in vivo* bacterial evolution during antibiotic pressure, (ii) antibiotic-mediated selection for a pre-existing intrinsically resistant subpopulation, and/or (iii) acquisition of an antibiotic resistant mutation from the external environment.

Here, we report on a case of persistent *S. epidermidis* bacteremia in a patient with an LVAD in which loss of susceptibility to both vancomycin and DAP arose during prolonged exposure to DAP. To the best of our knowledge, this is the first report to leverage whole genome sequencing (WGS) of isolates before and after antimicrobials to elucidate potential mechanisms of treatment-emergent DAP-NS within a patient host. We identified two mutations in the *WalK* gene from a single *S. epidermidis* strain (ST2) that became fixed after non-susceptible bloodstream isolates emerged, adding to the growing literature of the potential role of this gene in *S. epidermidis* DAP-NS.

## CASE PRESENTATION

A 49-year-old woman with a history of non-ischemic cardiomyopathy and previous biventricular intra-cardiac defibrillator device and LVAD placement underwent LVAD exchange owing to device malfunction. Six weeks later, a percutaneous drain was placed to evacuate a persistent post-operative fluid collection near the LVAD. Ten days thereafter (52 days after LVAD exchange), she presented with fever. Multiple blood cultures grew MDR-*S. epidermidis* (resistant to oxacillin, clindamycin, gentamicin, and trimethoprim-sulfamethoxazole) that was susceptible to vancomycin and DAP by E-test (Supplementary Table 1; Supplementary Methods). *S*taphylococcus *epidermidis* was isolated from the draining fluid, confirming the putative source of infection. Vancomycin was initiated immediately for VAD-related *S. epidermidis* bloodstream infection, but despite achieving therapeutic vancomycin serum concentrations, bacteremia persisted. On day 5, vancomycin was changed to DAP (6 mg/kg IV), the drain was exchanged, and blood cultures resulted with no growth within 48 h. Daptomycin was continued for 6 weeks (46 total days) at which time the drain was removed. The patient was transitioned to oral doxycycline for suppression until heart transplant; however, on day 10 of doxycycline, fevers and MDR-*S. epidermidis* bacteremia recurred despite complete resolution of the fluid collection. Daptomycin was re-initiated, but *S. epidermidis* continued to grow in blood cultures, and the new isolate demonstrated intermediate susceptibility to vancomycin (MIC = 8.0 μg/ml) and loss of DAP susceptibility (MIC = 2.0 μg/ml).

## CASE MANAGEMENT AND OUTCOME

Following the identification of a DAP-NS isolate, ceftaroline was initiated and bacteremia cleared within 48 h. However, eosinophilia and infusion-related chest pain after 20 days of ceftaroline prompted a change from ceftaroline to tigecycline. Surveillance blood cultures collected on day 6 of tigecycline again grew DAP-NS *S. epidermidis* (MIC = 4.0 μg/ml). Linezolid did not successfully clear bacteremia through 5-day monotherapy and another 4 days in combination with ceftaroline despite linezolid susceptibility (Supplementary Table 1). A combination of DAP–ceftaroline eventually led to sustained negative blood cultures and ultimately resolution of clinical symptoms. The patient was continued on DAP–ceftaroline without adverse events for more than 20 weeks until definitive VAD explant with heart transplantation was able to occur.

### Microbiologic evaluation

**Figure 1.**
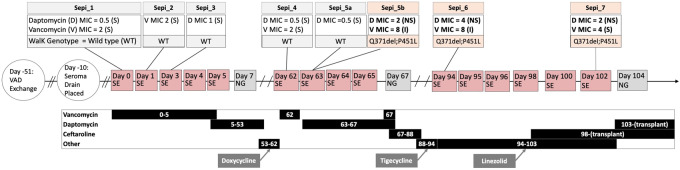
Timeline of clinical course: The clinical course of the patient (middle) with clinical events demarcated in circles while boxes indicate blood cultures (grey box: no growth; pink box: positive growth). The treatment regimen with days of antibiotic use is included (bottom) alongside the bloodstream isolate collection with susceptibility testing (top). VAD, ventricular assist device; SE, *Staphylococcus epidermidis*; NG, no growth; S, susceptible; NS, non-susceptible.

Vancomycin susceptibility testing (MIC) of the blood culture isolate from day 63 (Fig. 1) revealed both normal *S. epidermidis* growth around the E-test strip (Sepi_5a) and a smaller colony morphotype variant within the zone of inhibition of the E-test strip (Sepi_5b). To assess the colony heterogeneity, both colony morphotypes were sub-cultured, confirmed to be *S. epidermidis*, and vancomycin and DAP MICs were repeated on each morphotype separately and frozen separately (Supplementary Table 1). Given that distinct clones with different antimicrobial susceptibility patterns have recently been shown in a patient with VAD-related *S. aureus* endocarditis [14], we hypothesized that the presence of these two different morphotypes recovered from the same blood culture similarly indicated a mixed infection of two closely related strains.

To determine if there was a genotypic basis for the differing phenotypic colony morphology and susceptibility patterns, we performed pulse-field gel electrophoresis (PFGE) and WGS on the patient’s isolates that were frozen and stored during each round of antimicrobial susceptibility testing, including both colony morphologies from day 63 (Sepi_5a and Sepi_5b).

Analysis of the PFGE patterns showed that the small colony morphotype, Sepi_5b, had only a slight shift in one band compared with Sepi_5a that was indistinguishable from the pre-antibiotic reference Sepi_1 (Supplementary Fig. 2). Furthermore, these three isolates were distinct from two archived VAD-related *S. epidermidis* bacteremia isolates at our institution, used for comparison.

### Genomic analysis

DNA extraction was performed by picking multiple colonies from the pure subculture of each of the frozen bloodstream isolates to genetically characterize bloodstream isolates relevant to DAP exposure: seven time points, including both morphotypes from day 63 (Fig. 1). To capture as much genetic variation as possible, we performed short variant discovery and large structural variant discovery using a set of ‘raw’ (low stringency) and ‘filtered’ (high stringency) variants produced from reference guided mapping of paired-end WGS data (Supplementary Methods). In addition, we identified the species, multi-locus sequence type, resistance genes, and plasmids using a *de novo* assembly approach with the Centers for Genomic Epidemiology Bacterial Analysis Pipeline (Supplementary Fig. 1; Supplementary Methods) [15]. From these multiple approaches, we identified only two variants capable of completely segregating DAP-S isolates (Sepi_1, Sepi_2, Sepi_3, Sepi_4, and Sepi_5a) from DAP-NS isolates (Sepi_5b, Sepi_6, and Sepi_7; Supplementary Fig. 3). These sites encoded a three base-pair deletion (Q371del) and a missense mutation (P415L) in the WalK gene (NP_763574.1), respectively (Supplementary Fig. 4). Both variants were within the predicted kinase region (AA 384-602) of the protein (https://www.uniprot.org/uniprot/Q8CU87, July 2004, date last accessed) [13]. PROVEAN protein analysis indicated that the Q371del and P415L mutations had a deleterious predicted effect (Supplementary Table 2). Given the expected functional change from the mutation, we evaluated if structural changes in the protein could be predicted by homology modeling. The model indicates several disruptions in tertiary structures along the WalK protein when compared to wild-type protein, consistent with a non-neutral change in protein function (Supplementary Fig. 5).

## DISCUSSION

We report that a deletion of Q371 together with a single substitution mutation in *WalK* (P415L) arose coinciding with the loss of DAP susceptibility in an MDR-*S. epidermidis* (ST2) blood stream infection after prolonged DAP exposure. To the best our knowledge, this is the first report of *in vivo* induction of the *WalK* mutation in a CoNS clinical isolate that is absent in other heterogenous mutations [9] including previously reported MprF S295L substitutions [2, 16]. We found no evidence of sub-populations of different strains using both traditional microbiological methods and whole genome analysis. As a result, we conclude that *in vivo* mutation resulting from antibiotic pressure was the most likely explanation for clinical failure owing to the emergence of a DAP-NS *S. epidermidis* strain. In addition, a previous study has shown that *in vitro* selection for DAP-NS resulted in a V500F *WalK*, further supporting the relevance of Q371del/P415L for phenotypic loss of DAP susceptibility [13]. Finally, the Q371del/P415L mutation persisted after discontinuation of DAP (day 67), suggesting that the mutated strain may not incur a fitness-cost relevant to this clinical setting. Thus, our findings add to an increasing recognition of the role of mutations in the *WalK*/*R* sensing system and DAP resistance among CoNS (Table 1).

**Table 1.** Summary of *WalK* mutations and predicted effects

Isolate	Case complication	Antibiotic exposures	WalK (yycG) variant	Provean score	Predicted functional effect	Reference
*UNC* *S. epidermidis* (ST2)	Ventricular-assist device	Vancomycin × 5 days	Q371del, P415L	−11.313, −9.022	Deleterious, deleterious	This paper
		Daptomycin × 46 days				
		Doxycycline × 10 days				
*S. capitis* subsp. *Ureolyticus*	Ventricular-assist device	Vancomycin × 10 days	V220F	−1.505	Neutral	PMC6310605
		Daptomycin × 6 days				
*S. capitis* subsp. *Ureolyticus*	Aortic graft	Vancomycin × 14 days	N183I	−6.925	Deleterious	PMC6310605
*S. epidermidis*	*in vitro*	Daptomycin × 2 days	V500F	−3.058	Deleterious	PMC6395924
*S. capitis*	*in vitro*	Daptomycin × 11 days				PMC6395924
S. epidermidis*	Clinical isolate	Not available	G312S, D472H	2.28, 0.346	Neutral, neutral	PMC6395924
S. capitis*^†^	Clinical isolate	Not available	N48D	−1.05	Neutral	PMC6395924
S. capitis*^†^	Clinical isolate	Not available	N183I	−6.925	Deleterious	PMC6395924
S. capitis*^†^	Clinical isolate	Not available	G307D	−2.497	Neutral	PMC6395924
S. capitis*^†^	Clinical isolate	Not available	E573G	−6.526	Deleterious	PMC6395924
S. capitis*^†^	Clinical isolate	Not available				PMC6395924
*S. epidermidis*	Pacemaker	Vancomycin × 1 day	M428T	−5.983	Deleterious	PMC6408453
*S. epidermidis*	Deep wound extract pocket site infection	Vancomycin × 1 day	M428T	−5.983	Deleterious	PMC6408453

From a comprehensive literature review, we identified the previous reports of coagulase-negative *Staphylococcus* with daptomycin non-susceptible phenotypes and sequencing results. The isolate name, source (case complication), exposure to antibiotics, and the relevant mutations in the *WalK* gene are provided for each identified isolate. In addition, the PROVEAN score and predicted functional effect for each *WalK* variant are included. Given that PROVEAN estimates variant effects individually, the effects for the Q371del/P415L and G312S, D472H haplotypes cannot be estimated jointly. A subset of isolates underwent targeted Sanger sequencing and additional genome-wide effects are not available (*). Similarly, the co-occurrence of *WalK* and *WalR* mutations among a subset of *S. capitis* strains could not be determined from the manuscript (^†^).

How Q371del/P415L and V500F functional changes in *WalK* result in loss of DAP susceptibility is presently unknown. Similar to detergents, DAP is a bactericidal cell membrane-targeting lipopeptide. Perturbations in cell membrane events, including resistance to cell membrane depolarization and permeabilization, and reduced surface binding of DAP can lead to DAP resistance in *S. aureus* [17]. Interestingly, although overexpression of *WalKR* in *S. aureus* reduces susceptibility against vancomycin [18], the loss of *WalKR* expression resulting from mutations in regulatory genes leads to high resistance to cell lysis by Triton X-100 [11].

After prolonged exposure to a standard dose of DAP at 6 mg/kg IV, our patient developed a DAP-NS *S. epidermidis* infection with possible loss of vancomycin susceptibility. We cannot determine whether a higher initial dose of DAP may have averted DAP-NS evolution; however, increasing experience in the use of DAP for treatment of MRSA bloodstream infections suggests that the manufacturer-labeled dose may be inadequate, leading many experts to recommend doses of 8–10 mg/kg [19]. These considerations may be especially important for patients with an implanted cardiac device that cannot be removed, and when guideline-recommended adjuvant antibiotics such as aminoglycosides to promote bacterial clearance and/or rifampin to improve biofilm penetration [3] carry an unacceptably high risk of toxicity, as it is often the case of VAD-related bacteremia [1]. Further investigations are also needed to determine whether material-specific effects on biofilm production influence antibiotic resistance [20] in VAD-related infections, including regulation of *WalKR* [21].

Although we assumed that in our clinical setting, the Q371del/P415L mutation resulted in a low fitness cost, given its persistence even after the discontinuation of DAP treatment, it is possible that this persistence is not evidence of prosperity. In particular, the reversion of a deletion is highly unlikely and the Q371del/P415L mutation may represent an inescapable fitness in the absence of the DAP selective pressure. Future study with wild-type and isogenic knockout strains will be needed to elucidate this fitness landscape.

Increasing antimicrobial resistance among disease-associated *S. epidermidis* strains represents a global public health concern [2]. Given the current low incidence of DAP-R among *S. epidermidis* in the United States, DAP susceptibility is not universally reported by clinical microbiology laboratories and may often only be inferred. However, increasing reports of DAP-NS CoNS isolates raise the importance of vigilant monitoring and reporting of highly resistant isolates when they do occur. This may be of particular importance among patients with endovascular devices as a prior study found that only seven clones accounted for the majority of *S. epidermidis* infections in patients with VAD-related infection in the United States [22].

The utility of genomic and bioinformatic approaches for the surveillance, identification, and prediction of antibiotic resistance is being increasingly recognized as an aid or alternative to traditional clinical microbiology [23, 24]. In this manuscript, we combine these two disciplines together in a translational approach to elucidate the putative mechanism of antibiotic resistance observed in the clinic. In the future, similar translational approaches conducted in near real time would help to inform clinical practice by distinguishing the mechanism of resistance (e.g. *in vivo* evolution, selection of standing variation, and acquisition of resistance from the external environment), which thereby provides information for a tailored therapeutic response.

## CONCLUSION

Clinicians, hospital epidemiologists, and microbiology laboratories need to be aware of the potential for drug-resistance development on therapy. Daptomycin dosing is undergoing scrutiny for MRSA and Enterococcal infections, and our data support the consideration of using higher than manufacturer label (6 mg/kg) when treating high-burden endovascular device-related *S. epidermidis* bacteremia. Our findings suggest that DAP promoted the Q371del/P415L *WalK* mutation, but given that other environmental triggers, like detergents and divalent metals, may also regulate *WalKR*; further investigations into the range of hospital chemical inducers of reduced susceptibility to vancomycin and DAP are warranted.

## Supplementary Material

eoaa031_Supplementary_Data
