# Supplementary material for: Loss of daptomycin susceptibility in clinical Staphylococcus epidermidis infection coincided with variants in WalK
Source: Evol Med Public Health. 2020 Sep 7;2020(1):219–24. doi: 10.1093/emph/eoaa031 (PMC7658547; doi:10.1093/emph/eoaa031)
Supplement: eoaa031_Supplementary_Data [file eoaa031_supplementary_data.docx]

SUPPLEMENTARY MATERIAL

[IRB Statement 2](#_gjdgxs)

[Microbiology Methods and Results 2](#_30j0zll)

[Bacterial Culture and Susceptibility Testing 2](#_1fob9te)

[Pulse Field Gel Electrography 3](#_3znysh7)

[Genomic and Bioinformatic Methods 5](#_2et92p0)

[Isolate Sequencing Library Preparation 5](#_tyjcwt)

[Reference Guided Alignment and Variant Discovery 5](#_3dy6vkm)

[*de novo* Assembly and Gene Identification 6](#_1t3h5sf)

[Protein Function Analysis and Structure Homology Modeling 7](#_4d34og8)

[Analyses & Reproducibility 7](#_2s8eyo1)

[Genomic and Bioinformatic Results 8](#_17dp8vu)

[Short Variants 8](#_3rdcrjn)

[Structural Variants 10](#_26in1rg)

[Resistance Genes and Plasmids 10](#_lnxbz9)

[Protein Function Analysis and Homology Modeling 12](#_35nkun2)

[REFERENCES 13](#_1ksv4uv)

## IRB Statement

This research was approved by the University of North Carolina Institutional Review Board. Patient consent was acquired prior to the study.

## Microbiology Methods and Results

### Bacterial Culture and Susceptibility Testing

All microbial testing procedures were performed according to Clinical Laboratory Improvement Amendments (CLIA) standards. Blood culture bottles were collected and incubated on the BD BacTec FX system, and all positive blood culture bottles were plated for culture. Bacterial growth was identified by Matrix-Assisted Laser Desorption/Ionization-Time of Flight (MALDI-TOF, bioMérieux), and antimicrobial susceptibility testing (AST) was performed via Kirby-Bauer disc diffusion and E-test to determine MICs. In order to capture potential heterogeneity of the bacterial population, AST was performed by picking ≥3 isolated colonies from the blood culture plates to make a suspension (McFarland standard), then plated as a lawn on the AST plate. Disks and E-tests were applied, plates were incubated (37°C) overnight, and zones of inhibition were read and interpreted the next day. AST interpretations were determined according to the 2018 Clinical Laboratory Standards Institute (CLIS) M100 guidelines.

All positive blood cultures in which AST had been performed, were archived as frozen stocks as part of routine patient care at the University of North Carolina Hospital and the microbiology laboratory from the referring hospital (RH) where the patient initially presented. Frozen stocks were made by swiping a portion of the lawn of bacterial growth from the AST plate. As a result, these frozen isolates represented time-points before, during, and after relevant antibiotic pressure (**Figure 1**).

The first isolate obtained during the incident bacteremia prior to antibiotic treatment (Sepi_1) served as a pre-antibiotic exposure reference (vancomycin MIC = 2.0 µg/mL, daptomycin MIC = 0.5 µg/mL). Additionally, we subcultured the first isolate at UNC-CH after 1 day of vancomycin (Sepi_2) (vancomycin MIC = 2.0 µg/mL) and the last isolate after 5 days of vancomycin (before daptomycin) (Sepi_3) (vancomycin MIC = 2.0 µg/mL, daptomycin MIC = 1 µg/mL). Similarly, for the first relapse bacteremia we subcultured the first isolate (Sepi_4) that was present during doxycycline exposure (vancomycin MIC = 2.0 µg/mL, daptomycin MIC = 0.5 µg/mL), and the first positive blood culture at UNC after 1 dose of daptomycin (Sepi_5) (vancomycin MIC = 8.0 µg/mL, daptomycin MIC = 2.0 µg/mL). For the second relapse, we subcultured from breakthrough blood cultures collected on tigecycline (Sepi_6) (vancomycin MIC = 8.0 µg/mL, daptomycin MIC = 4.0 µg/mL) and after 9 days of linezolid (Sepi_7) (vancomycin MIC = 4.0 µg/mL, daptomycin MIC = 2.0 µg/mL) (**Supplementary Table 1**). Interestingly, subculture of Sepi_5 revealed two morphotypes (Sepi_5a and Sepi_5b) resulting in 8 total isolates. Susceptibility testing of Sepi_5a (regular morphotype) resulted with a daptomycin MIC = 0.5 µg/mL whereas Sepi_5b (small variant morphotype) resulted in daptomycin MIC = 2.0 µg/mL, consistent with a mixed population of *S. epidermidis* on this day. All other sub-cultures yielded a single morphotype.

Prior to PFGE and WGS, all subcultures of the isolates from the frozen stocks were confirmed to be pure *S. epidermidis* isolates with colony morphology that matched the original AST culture.

### Pulse Field Gel Electrography

DNA extraction, plug preparation, and electrophoresis parameters were performed according to protocol. DNA digestion of the *S. epidermidis* isolates was done using the *Sma*I enzyme. Banding patterns and degree of genetic relatedness was determined by visual analysis and compared to archived clinical strains of VAD-related *S. epidermidis* isolates from the same institution (UNC_1 and UNC_2) .


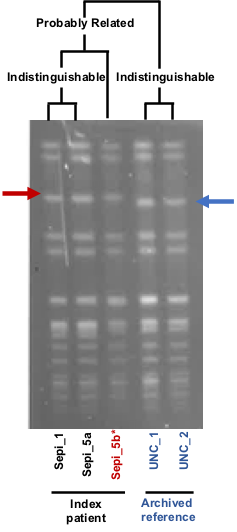


**Supplementary Figure 1 - Pulse-field gel electrophoresis (PFGE):** Three representative *S. epidermidis* isolates from the index patient in this report (Sepi_1, Sepi_5a, Sepi_5b) and two *S. epidermidis* isolates from a clinical archive (UNC_1, UNC_2). Only Sepi_5b showed reduced susceptibility to vancomycin and daptomycin. The third band from the top (arrows) showed slight elevation in Sepi_5b and depression in UNC_1 and UNC_2 compared with the Sep1_1 reference isolate. No other significant differences were seen by PFGE.

| **Clinical Course** | | | | | | | | |
| --- | --- | --- | --- | --- | --- | --- | --- | --- |
| **Isolate Timepoints** | 1* | 2 | 3 | 4 | 5 | 6 | 7 |  |
| **Day of Clinical Infection** | 0 | 1 | 3 | 62 | 63 | 94 | 102 |  |
| **Antibiotic Susceptibility Interpretation** | | | | | | | | |
| **Ceftaroline** | - | - | - | - | S | S | S |  |
| **Clindamycin** | - | R | - | - | R | R | R |  |
| **Daptomycin** | S (MIC=0.5) | - | S (MIC=1) | S (MIC=0.5) | NS (MIC=2) | NS (MIC=4) | NS (MIC=2) |  |
| **Doxycycline** | - | S | - | - | S | S | S |  |
| **Erythromycin** | - | R | - | - | R | R | R |  |
| **Gentamicin** | - | R | - | - | R | R | R |  |
| **Linezolid** | - | - | - | - | S | S | S |  |
| **Oxacillin** | - | R | - | - | R | R | R |  |
| **Rifampin** | - | S | - | - | - | S | - |  |
| **Tigecycline** | - | - | - | - | MIC=1 | MIC=0.5 | - |  |
| **SMX/TMP˚** | - | R | - | - | R | R | R |  |
| **Vancomycin** | S (MIC=2) | S (MIC=2) | - | S (MIC=2) | I (MIC=8) | I (MIC=8) | S (MIC=4) |  |

**Supplementary Table 1 - Susceptibility Panel on *S. epidermidis* Isolates from Positive Blood Cultures across 3 episodes of bacteremia:** Susceptibility testing indicates that daptomycin non-susceptibility emerged at time points 5-7. All susceptibilities performed by disk diffusion & E-test except OSH isolates. All MIC’s reported in µg/mL. *Abbreviations:* (-) test not performed; **˚**SMX/TMP: Sulfamethoxazole/Trimethoprim; *****Susceptibility testing at outside hospital (OSH) performed via MicroScan; NS: non-susceptible.

## Genomic and Bioinformatic Methods

### Isolate Sequencing Library Preparation

DNA from the bacterial colonies were sheared with a Covaris E220 instrument and prepared for sequencing with the KAPA Hyper Prep Kit (Roche Sequencing©, Pleasanton, CA). Indexed samples were then pooled in equimolar amounts into a final library. This library was then sequenced on a MiSeq2000 2x75 pair-end run (Illumina®, San Diego, CA) at the University of North Carolina High Throughput Sequencing Facility.

### Reference Guided Alignment and Variant Discovery

Raw reads first underwent adapter trimming using `cutadapt` (v1.16). Trimmed reads were then aligned to *S. epidermidis* ATCC 12228 nuclear genome (NC_004461.1; downloaded November 15, 2018; <https://www.ncbi.nlm.nih.gov/genome/155?genome_assembly_id=299299>) using `bwa-mem` (v0.7.17). Alignments were then deduplicated and annotated with mate-tags using `samblaster` (v0.1.24). Resulting alignments were assessed for depth of coverage at the genomic and base-level using `bedtools genomecov` and quality using Genome Analysis Toolkit (GATK) `CallableLoci` (v3.8.0). We defined a loci as “callable” if the site had a mapping quality of at least 55 and a base quality of at least 30 (MQ ≥ 55, BQ ≥ 30).

We then performed short variant discovery for each clinical isolate using GATK `HaplotypeCaller` as haploids (`-ploidy 1`) followed by joint genotyping across all isolates with GATK `GenotypeGVCFs` (v4.0.3) [[1]](https://paperpile.com/c/U34NJT/YPZFc). We then limited our analysis to biallelic sites under the assumption that multiallelic sites were due to sequencing error. Analyses focused on segregating sites, or loci that differed among isolates.

In order to capture as much variation as possible, we performed a “raw” analysis of the short variants and a “filtered” analysis of the short variants. These analyses are expected to be highly sensitive and highly specific, respectively.

For the raw short variant analysis, in addition to the biallelic and segregating site criteria, we excluded loci if more than three isolates lacked coverage at the given site. Segregating sites were identified from the within-sample non-referent allele frequencies (WSNRAF) instead of the genotype calls to maximize sensitivity. We identified segregating sites by determining which sites had at least a 5% difference in the maximum and minimum WSNRAF among the isolates.

For the filtered short variant analysis, in addition to the biallelic and segregating site criteria, we excluded variants with a mapping-quality less than 55 (MQ < 55) or a strands odds ratio of greater than two (SOR > 2). In addition, we masked variants that fell within low complexity regions. Low complexity regions were identified by aligning the reference genome to itself using the `nucmer` tool from MUMmer (v3.23.3). Any region that had more than 90% similarity to its own genome was masked by excluding an interval 50 base-pairs upstream and 50 bases-downstream downstream of the repeat region. We identified segregating sites based on the genotype calls, such that at least one isolate had a unique genotype (i.e. for biallelic haplotypes “0: vs. “1”). Variants were annotated using `snpEff` (v4.3t) and GATK `VariantAnnotator` (v3.8.0) with a pre-build database that was exported along with the `snpEff` (Staphylococcus_epidermidis_atcc_12228).

Large variant discovery was limited to insertions and deletions (INDELs) and was performed with `smoove` (v0.2.3) which wraps several tools, including `lumpy-sv`, `SVTyper`, and `duphold` [[2–4]](https://paperpile.com/c/U34NJT/8r8Ny+GrpkE+yyN2o). As above, we first considered all INDELs and then a filtered set of structural variants that had coverage in at least five clinical isolates. When quality filters for structural variants were considered, we excluded duplications with less than or equal to 1.3-fold coverage relative to regions with similar GC-content (DHBFC ≤ 1.3) and deletions with greater than or equal to 0.7-fold coverage relative to the rest of the genome (DHFFC ≥ 0.7; note, one nuclear chromosome) on an isolate-by-isolate basis [[4]](https://paperpile.com/c/U34NJT/yyN2o). Finally, we excluded INDELs that fell within regions of low-complexity identified above. As above, we identified segregating sites based on the genotype calls.

### *de novo* Assembly and Gene Identification

Raw reads were passed to the `Bacterial Analysis Pipeline` (v1.2.1) using the `KmerFinder` (v3.0), `MLST` (v2.0), and `ResFinder` (v2.1) submodules [[5–8]](https://paperpile.com/c/U34NJT/SGGt3+bBEYN+yLxlP+Xd6Tj). Briefly, we used the pipeline to assemble the raw reads (internal script, v2.0) specifying the Illumina®️ paired-end sequencing flag (`--Asp Illumina --Ast paired`). In parallel, the raw reads were used to identify the species using a k-mer based approach (`KmerFinder`). Assembled sequences were then queried against a series of bacterial databases using the `ncbi-blast` suite (v2.2.29-3) to identify predefined alleles (`MLST`) and resistance genes (`ResFinder`) with default settings. The `Bacterial Analysis Pipeline` was cloned from the Centers for Genomic Epidemiology (CGE) Bitbucket®️ as a docker image (<https://bitbucket.org/genomicepidemiology/cge-tools-docker/src/master/>) on October 24, 2018. Bacterial databases downloaded from the CGE repository (ftp://[ftp.cbs.dtu.dk/public/CGE/databases](http://ftp.cbs.dtu.dk/public/CGE/databases)) on October 30, 2018.

Separately, we used `PlasmidFinder` (v2.0) with the `kma` (v1.2.3t) algorithm to rapidly query raw reads against a Gram-Positive plasmid database (`--databases "gram_positive" `) database and identify plasmid matches [[9,10]](https://paperpile.com/c/U34NJT/EMRuU+cEmDS). The `PlasmidFinder` docker image (<https://bitbucket.org/genomicepidemiology/plasmidfinder/src>) was cloned from the CGE Bitbucket®️ on August 21, 2019 and the plasmid-finder gram positive plasmid database was built on June 21, 2019 (<https://bitbucket.org/genomicepidemiology/plasmidfinder_db.git>).


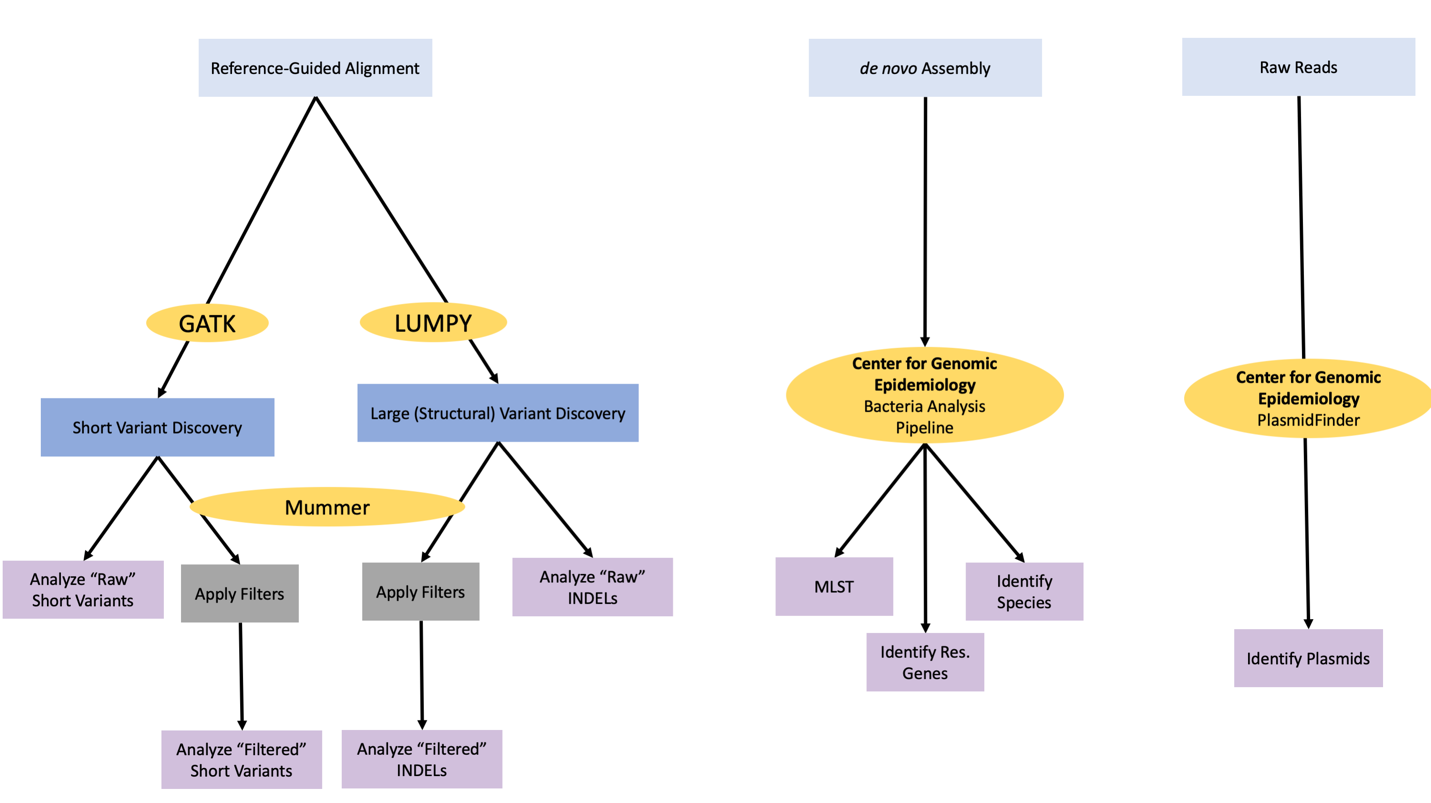


**Supplementary Figure 2 - Bioinformatic Workflow:** Paired-end whole genome sequencing data was assessed using short variant discovery, structural variant discovery, *de novo* assembly and Kmer-alignment based approaches. For variant discovery, both “raw” and “filtered” analyses were considered.

### Protein Function Analysis and Structure Homology Modeling

Protein function was analyzed using PROVEAN (online access: [http://provean.jcvi.org/](http://provean.jcvi.org/index.php)) with default settings and the amino acid sequences for wild type *WalK* (AAO03616.1) [[11]](https://paperpile.com/c/U34NJT/FNJZg). The default PROVEAN score threshold of -2.5 was used to classify functional effect predictions as deleterious or neutral (default setting).

The amino acid sequences for wild type *WalK* (AAO03616.1) and variant WalK sequences identified in our study (Q371del/P415L) were used to model the three-dimensional structure of the protein [[12]](https://paperpile.com/c/U34NJT/l8a2r). Homology models were developed using SWISS-Model [[13,14]](https://paperpile.com/c/U34NJT/fvpfq+tXxr2). The model was constructed using the x-ray diffraction structure of a histidine kinase (4I5S, B chain: <https://www.rcsb.org/structure/4i5s>) [[15]](https://paperpile.com/c/U34NJT/hLwDo). The structures were superimposed and visualized using CHIMERA [[16]](https://paperpile.com/c/U34NJT/I6QwA).

### Analyses & Reproducibility

Filtering and plotting were primarily performed using the R-software (v3.5.2) and the `tidyverse` (v1.2.1), `vcfR` (v1.8.0), and `seqinr` (v3.4.5) packages [[17–19]](https://paperpile.com/c/U34NJT/gyMpI+hcAab+2EfnU). All scripts and code used to generate this data are available on Github: nickbrazeau/Sepi_Res_CaseStudy. All sequencing data is publicly available via the NCBI short read archive (accession numbers: PRJNA563309).

## Genomic and Bioinformatic Results

Across the eight isolates, we achieved 25-fold coverage in at least 95.6% of the genome (overall mean coverage depth ranged from 48.8- to 119.1-bases). In addition, based on our thresholds for a “callable loci”, we found that at least 93.3% of the genome was considered high-quality. Overall, this indicates that we successfully sequenced these clinical isolates to an appropriate depth and quality.

### Short Variants

We identified 9,491 short variants when aligning to the *S. epidermidis* ATCC 12228 reference genome. Among these short variants, 9,457 were biallelic. We identified 8,892 single- and multi-nucleotide variants and 565 (short) insertions/deletions among the biallelic variants. For the raw analysis, we only identified as 762 segregating sites by the WSNRAF among the biallelic loci. Among these 762 sites, only two variants differentiated the DAP-S isolates (Sepi_1, Sepi_2, Sepi_3, Sepi_4, and Sepi_5a) and the DAP-NS isolates (Sepi_5b, Sepi_6, and Sepi_7). These two variants both fell within the *WalK* gene (NP_763574.1), and encoded a three base-pair deletion (Q371del) and a missense mutation (P415L), respectively.

When considering the filtered analysis, we identified 7,778 biallelic short variants that passed our thresholds. Among these high-quality short variants, we identified 21 segregating sites. As above, the only two variants that separated the DAP-S and DAP-NS clinical phenotypes were Q371del and P415L, respectively (**Supplementary Figure 3**). Both of these mutations fall within the predicted kinase region (AA 384-602) of the WalK protein (<https://www.uniprot.org/uniprot/Q8CU87>) [[12]](https://paperpile.com/c/U34NJT/l8a2r).

**
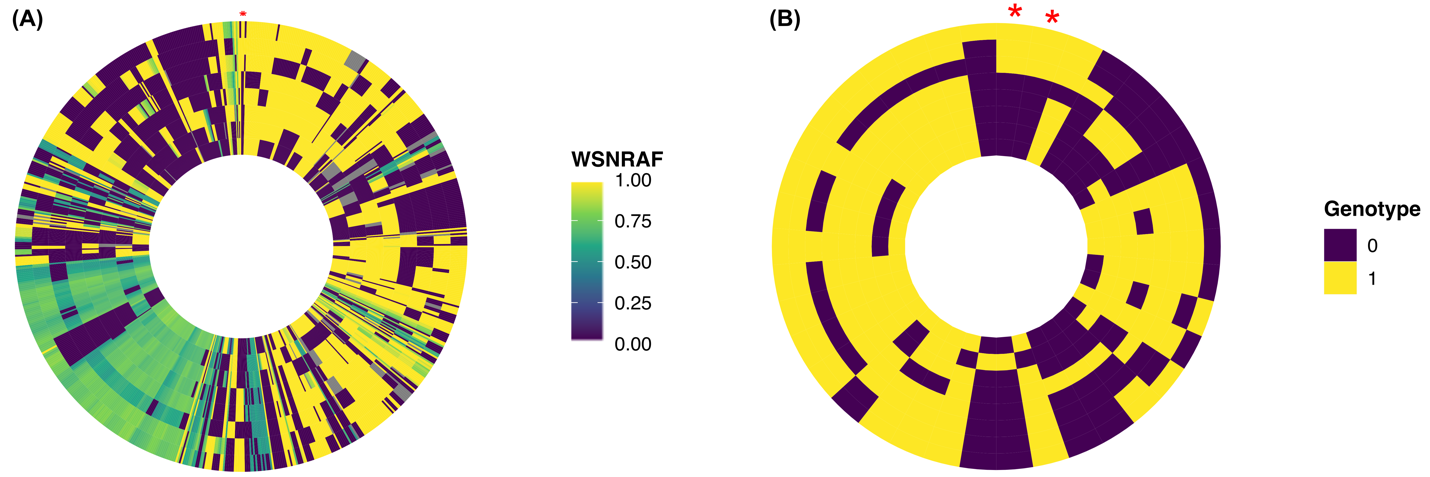
**

**Supplementary Figure 3 - Allele Distributions among Clinical Isolates:** Clinical isolates are ordered as rows by date-of-collection with the first isolate placed closest to the center. (**A**) The within-sample non-referent allele-frequency spectrum (WSNRAF) for the “raw” segregating sites among the clinical isolates. (**B**) The genotype-calls for the “filtered” segregating sites among the clinical isolates. Segregating sites are concatenated along the x-axis with respect to position along the nuclear genome. The differentiating sites among the daptomycin-sensitive phenotype (MIC ≤ 1 µg/mL; Sepi_1, Sepi_2, Sepi_3, Sepi_4, Sepi_5a) and the daptomycin-resistant phenotype (MIC ≥ 2 µg/mL; Sepi_5b, Sepi_6, and Sepi_7) are indicated by a red asterisk. In both cases, although there may be some distinguishing sites among each isolate, only two sites can differentiate the clinical phenotypes.


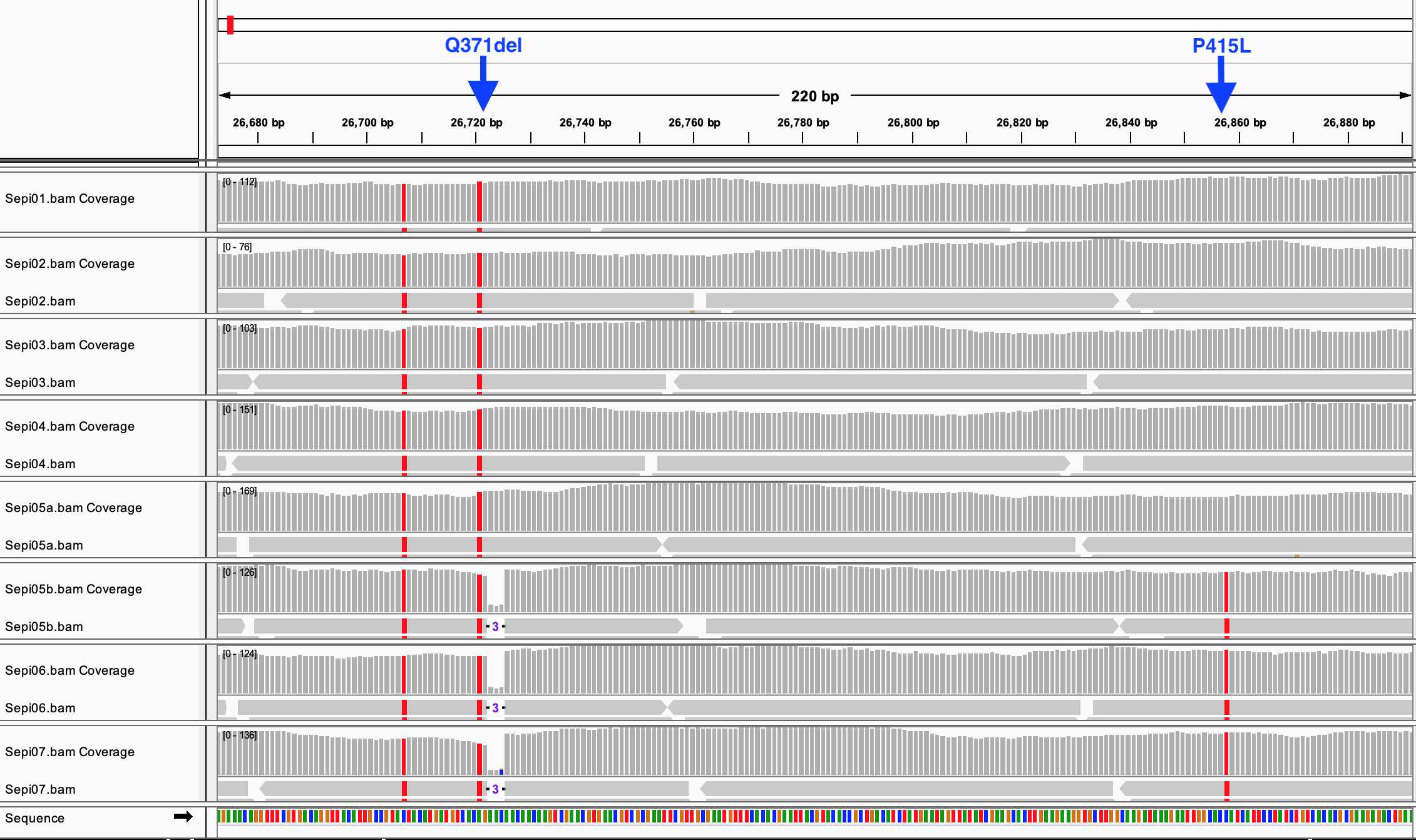


**Supplementary Figure 4 - Visualization of Differentiating Variants:** When visualizing the raw alignments, the Q371del and P415L missense mutation plainly differentiate the daptomycin-sensitive phenotype (Sepi_1, Sepi_2, Sepi_3, Sepi_4, Sepi_5a) and the daptomycin-resistant phenotype (Sepi_5b, Sepi_6, and Sepi_7).

### Structural Variants

We identified 58 large structural variants among the clinical isolates, of which only 25 loci were identified as segregating sites. Similarly, when we applied our filters to the segregating sites, only four loci passed our thresholds. Among both the raw and the filtered structural variants, no sites differentiated the DAP-S and DAP-NS clinical phenotypes.

### Resistance Genes and Plasmids

Throughout the entire course of the infection, all isolates were typed as the ST2-strain. Similarly, the aac(6')-aph(2''), aadD, blaZ, erm(C), fosB, mecA resistance genes associated with aminoglycoside-resistance, aminoglycoside-resistance, beta-lactam resistance, macrolide resistance, fosfomycin resistance, and beta-lactam resistance, respectively, remained unchanged throughout the course of infection. Finally, plasmids also remained unchanged throughout the course of infection.

| **Isolate** | **Genome Size** | **Contigs** | **n50** | **Species** | **MLST** | **MLST Genes** | **Resistance Genes** | **Plasmids** |
| --- | --- | --- | --- | --- | --- | --- | --- | --- |
| Sepi_1 | 2602734 | 111 | 96277 | Staphylococcus epidermidis | Staphylococcus epidermidis[ST2] | arcC_7, aroE_1, gtr_2, mutS_2, pyrR_4, tpiA_1, yqiL_1 | aac(6')-aph(2''), aadD, blaZ, erm(C), fosB, mecA | rep10-ORF(pNE131), rep10-repL(pDLK1), rep22-repB(pUB110), repUS12-rep(pUB110), repUS23-repA(SAP099B) |
| Sepi_2 | 2601513 | 126 | 96269 | Staphylococcus epidermidis | Staphylococcus epidermidis[ST2] | arcC_7, aroE_1, gtr_2, mutS_2, pyrR_4, tpiA_1, yqiL_1 | aac(6')-aph(2''), aadD, blaZ, erm(C), fosB, mecA | rep10-ORF(pNE131), rep10-repL(pDLK1), rep22-repB(pUB110), repUS12-rep(pUB110), repUS23-repA(SAP099B) |
| Sepi_3 | 2602114 | 110 | 89513 | Staphylococcus epidermidis | Staphylococcus epidermidis[ST2] | arcC_7, aroE_1, gtr_2, mutS_2, pyrR_4, tpiA_1, yqiL_1 | aac(6')-aph(2''), aadD, blaZ, erm(C), fosB, mecA | rep10-ORF(pNE131), rep10-repL(pDLK1), rep22-repB(pUB110), repUS12-rep(pUB110), repUS23-repA(SAP099B) |
| Sepi_4 | 2602551 | 113 | 99647 | Staphylococcus epidermidis | Staphylococcus epidermidis[ST2] | arcC_7, aroE_1, gtr_2, mutS_2, pyrR_4, tpiA_1, yqiL_1 | aac(6')-aph(2''), aadD, blaZ, erm(C), fosB, mecA | rep10-ORF(pNE131), rep10-repL(pDLK1), rep22-repB(pUB110), repUS12-rep(pUB110), repUS23-repA(SAP099B) |
| Sepi_5a | 2602232 | 118 | 96269 | Staphylococcus epidermidis | Staphylococcus epidermidis[ST2] | arcC_7, aroE_1, gtr_2, mutS_2, pyrR_4, tpiA_1, yqiL_1 | aac(6')-aph(2''), aadD, blaZ, erm(C), fosB, mecA | rep10-ORF(pNE131), rep10-repL(pDLK1), rep22-repB(pUB110), repUS12-rep(pUB110), repUS23-repA(SAP099B) |
| Sepi_5b | 2604077 | 123 | 99559 | Staphylococcus epidermidis | Staphylococcus epidermidis[ST2] | arcC_7, aroE_1, gtr_2, mutS_2, pyrR_4, tpiA_1, yqiL_1 | aac(6')-aph(2''), aadD, blaZ, erm(C), fosB, mecA | rep10-ORF(pNE131), rep10-repL(pDLK1), rep22-repB(pUB110), repUS12-rep(pUB110), repUS23-repA(SAP099B) |
| Sepi_6 | 2599470 | 123 | 96261 | Staphylococcus epidermidis | Staphylococcus epidermidis[ST2] | arcC_7, aroE_1, gtr_2, mutS_2, pyrR_4, tpiA_1, yqiL_1 | aac(6')-aph(2''), aadD, blaZ, erm(C), fosB, mecA | rep10-ORF(pNE131), rep10-repL(pDLK1), rep22-repB(pUB110), repUS12-rep(pUB110), repUS23-repA(SAP099B) |
| Sepi_7 | 2600834 | 114 | 89513 | Staphylococcus epidermidis | Staphylococcus epidermidis[ST2] | arcC_7, aroE_1, gtr_2, mutS_2, pyrR_4, tpiA_1, yqiL_1 | aac(6')-aph(2''), aadD, blaZ, erm(C), fosB, mecA | rep10-ORF(pNE131), rep10-repL(pDLK1), rep22-repB(pUB110), repUS12-rep(pUB110), repUS23-repA(SAP099B) |

**Supplementary Table 2:** Analyses from the Center for Genomic Epidemiology Toolkit. The size of the assembly (Genome Size), the number of contigs from the assembly (Num. Contigs), and the assembly N50 for assembly quality assurance. In addition, the species composition of each isolate identified by `KmerFinder` (Species) and the multilocus sequence typing (MLST) results and MLST genes (MLST, MLST Genes) from `MLST` are shown. Finally, resistance genes from `ResFinder` and plasmids from `PlasmidFinder` are detailed for each isolate.

### Protein Function Analysis and Homology Modeling

Protein analysis indicated that the Q371del variant and the P415L variant both were predicted to have a deleterious effect on protein function (deleterious versus neutral; **Table 1**). This deleterious effect is consistent with the predicted functional shifts for the V500F variant, which has previously been shown to cause the daptomycin non-susceptible phenotype [[12]](https://paperpile.com/c/U34NJT/l8a2r). We were able to successfully model approximately half of the protein (wildtype, amino acids: 209-599; mutant, amino acids: 225-599; full protein length: 610 amino acids). Relative to the wild type WalK protein, the WalK protein (Q371del/P415L) lost several tertiary protein structures (**Supplementary Figure 2**). Loss of these tertiary structures are consistent with the conclusion that the Q371del/P415 variants have a non-neutral -- and potentially deleterious effect -- on the WalK protein structure. However, further work with a “knock-in” bacteria model is needed to confirm functional effects.

**
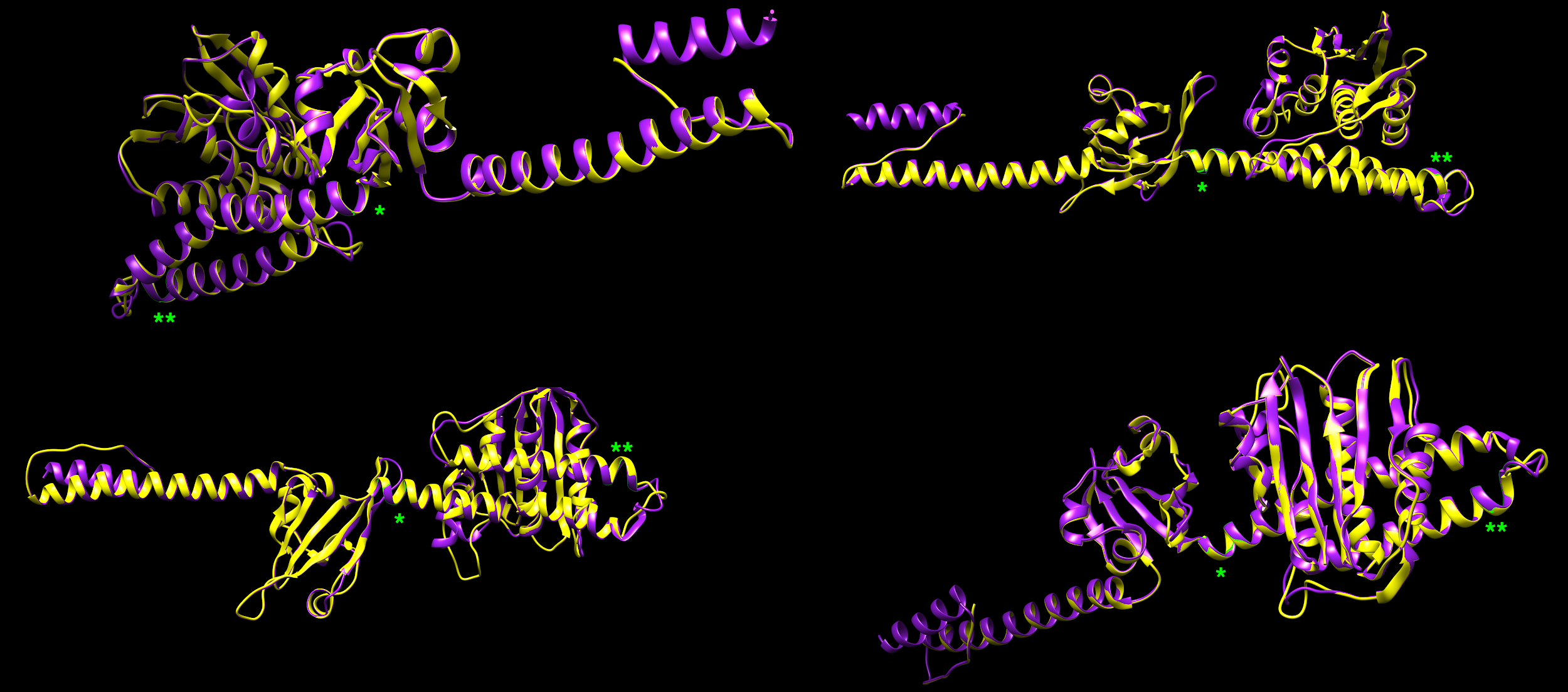
**

**Supplementary Figure 5 - Four Perspectives of the 3D WalK Protein Model.** Although the wild type (purple) and mutant (yellow) WalK protein from our study are largely homologous, the mutant WalK protein lacks several tertiary structures relative to the wild type protein -- namely, several alpha helices are lost. The locations of the Q371del variant (*) and the P415L (**) variants are indicated throughout the different views of the 3D structure of the WalK protein.

## REFERENCES

[1. Poplin R, Ruano-Rubio V, DePristo MA *et al.* Scaling accurate genetic variant discovery to tens of thousands of samples. *bioRxiv* 2017:201178.](http://paperpile.com/b/U34NJT/YPZFc)

[2. Layer RM, Chiang C, Quinlan AR *et al.* LUMPY: a probabilistic framework for structural variant discovery. *Genome Biol* 2014;**15**:R84.](http://paperpile.com/b/U34NJT/8r8Ny)

[3. Chiang C, Layer RM, Faust GG *et al.* SpeedSeq: ultra-fast personal genome analysis and interpretation. *Nat Methods* 2015;**12**:966–8.](http://paperpile.com/b/U34NJT/GrpkE)

[4. Pedersen BS, Quinlan AR. Duphold: scalable, depth-based annotation and curation of high-confidence structural variant calls. *Gigascience* 2019;**8**, DOI:](http://paperpile.com/b/U34NJT/yyN2o) [10.1093/gigascience/giz040](http://dx.doi.org/10.1093/gigascience/giz040)[.](http://paperpile.com/b/U34NJT/yyN2o)

[5. Thomsen MCF, Ahrenfeldt J, Cisneros JLB *et al.* A Bacterial Analysis Platform: An Integrated System for Analysing Bacterial Whole Genome Sequencing Data for Clinical Diagnostics and Surveillance. *PLoS One* 2016;**11**:e0157718.](http://paperpile.com/b/U34NJT/SGGt3)

[6. Larsen MV, Cosentino S, Rasmussen S *et al.* Multilocus sequence typing of total-genome-sequenced bacteria. *J Clin Microbiol* 2012;**50**:1355–61.](http://paperpile.com/b/U34NJT/bBEYN)

[7. Zankari E, Hasman H, Cosentino S *et al.* Identification of acquired antimicrobial resistance genes. *J Antimicrob Chemother* 2012;**67**:2640–4.](http://paperpile.com/b/U34NJT/yLxlP)

[8. Hasman H, Saputra D, Sicheritz-Ponten T *et al.* Rapid whole-genome sequencing for detection and characterization of microorganisms directly from clinical samples. *J Clin Microbiol* 2014;**52**:139–46.](http://paperpile.com/b/U34NJT/Xd6Tj)

[9. Carattoli A, Zankari E, García-Fernández A *et al.* In silico detection and typing of plasmids using PlasmidFinder and plasmid multilocus sequence typing. *Antimicrob Agents Chemother* 2014;**58**:3895–903.](http://paperpile.com/b/U34NJT/EMRuU)

[10. Clausen PTLC, Aarestrup FM, Lund O. Rapid and precise alignment of raw reads against redundant databases with KMA. *BMC Bioinformatics* 2018;**19**:307.](http://paperpile.com/b/U34NJT/cEmDS)

[11. Choi Y, Sims GE, Murphy S *et al.* Predicting the functional effect of amino acid substitutions and indels. *PLoS One* 2012;**7**:e46688.](http://paperpile.com/b/U34NJT/FNJZg)

[12. Jiang J-H, Dexter C, Cameron DR *et al.* Evolution of Daptomycin Resistance in Coagulase-Negative Staphylococci Involves Mutations of the Essential Two-Component Regulator WalKR. *Antimicrob Agents Chemother* 2019;**63**, DOI:](http://paperpile.com/b/U34NJT/l8a2r) [10.1128/AAC.01926-18](http://dx.doi.org/10.1128/AAC.01926-18)[.](http://paperpile.com/b/U34NJT/l8a2r)

[13. Waterhouse A, Bertoni M, Bienert S *et al.* SWISS-MODEL: homology modelling of protein structures and complexes. *Nucleic Acids Res* 2018;**46**:W296–303.](http://paperpile.com/b/U34NJT/fvpfq)

[14. Bienert S, Waterhouse A, de Beer TAP *et al.* The SWISS-MODEL Repository-new features and functionality. *Nucleic Acids Res* 2017;**45**:D313–9.](http://paperpile.com/b/U34NJT/tXxr2)

[15. Wang C, Sang J, Wang J *et al.* Mechanistic insights revealed by the crystal structure of a histidine kinase with signal transducer and sensor domains. *PLoS Biol* 2013;**11**:e1001493.](http://paperpile.com/b/U34NJT/hLwDo)

[16. Pettersen EF, Goddard TD, Huang CC *et al.* UCSF Chimera—a visualization system for exploratory research and analysis. *J Comput Chem* 2004;**25**:1605–12.](http://paperpile.com/b/U34NJT/I6QwA)

[17. Wickham H. Tidyverse: Easily install and load’tidyverse’packages. *R package version* 2017;**1**.](http://paperpile.com/b/U34NJT/gyMpI)

[18. Knaus BJ, Grünwald NJ. vcfr: a package to manipulate and visualize variant call format data in R. *Mol Ecol Resour* 2017;**17**:44–53.](http://paperpile.com/b/U34NJT/hcAab)

[19. Charif D, Lobry JR. SeqinR 1.0-2: A Contributed Package to the R Project for Statistical Computing Devoted to Biological Sequences Retrieval and Analysis. In: Bastolla U, Porto M, Roman HE, et al. (eds.). *Structural Approaches to Sequence Evolution: Molecules, Networks, Populations*. Berlin, Heidelberg: Springer Berlin Heidelberg, 2007, 207–32.](http://paperpile.com/b/U34NJT/2EfnU)
